# *Which Way?* Indigenous-led Smoking Cessation Care: Knowledge, Attitudes and Practices of Aboriginal and Torres Strait Islander Health Workers and Practitioners – A National Cross-sectional Survey

**DOI:** 10.1093/ntr/ntac256

**Published:** 2022-11-05

**Authors:** Michelle Kennedy, Hayley Longbottom, Amanual Mersha, Raglan Maddox, Karl Briscoe, Paul Hussein, Shanell Bacon, Yael Bar-Zeev

**Affiliations:** College of Health Medicine and Wellbeing, The University of Newcastle, Callaghan, NSW, Australia; Hunter Medical Research Institute, New Lambton Heights, NSW, Australia; Waminda- South Coast Women’s Health and Welfare Aboriginal Coorporation, Nowra, NSW, Australia; College of Health Medicine and Wellbeing, The University of Newcastle, Callaghan, NSW, Australia; Hunter Medical Research Institute, New Lambton Heights, NSW, Australia; National Centre for Epidemiology and Public Health, The Australian National University, Canberra, Australia; National Association of Aboriginal and Torres Strait Islander Health Workers and Practitioners, Phillip, ACTAustralia; Yerin - Eleanor Duncan Aboriginal Health Centre, Wyong, NSW, Australia; Nunyara Aboriginal Health Unit, Gosford, NSW, Australia; Braun School of Public Health and Community Medicine, Faculty of Health, Hebrew University of Jerusalem, Israel

## Abstract

**Introduction:**

Tobacco is the leading preventable cause of morbidity and mortality for Aboriginal and Torres Strait Islander people. Accordingly, the provisions of culturally safe and effective smoking cessation strategies are crucial. While previous research has suggested Aboriginal Health Workers/Practitioners are well placed to provide smoking cessation care, no research to date has explored the workforce knowledge, attitudes and practices in offering best practice cessation care.

**Methods:**

A cross-sectional study was conducted among Aboriginal Health Workers/Practitioners from June to September 2021. Descriptive and inferential statistics were conducted to examine participant characteristics, provision of smoking cessation care, and explore the factors associated with smoking cessation care.

**Results:**

Out of 1052 registered Aboriginal Health Workers/Practitioners, 256 participants completed the full survey (24.3%). Smoking cessation counseling was always provided by 41.9%; provided some of the time by 42.4%, and never provided by 12.9%. Combination NRT and Quitline referral were always offered by 23.1% and 44.9% of participants, respectively. Those that received training, felt smoking cessation care was part of their role, and were based in Aboriginal Community Controlled Health Organization were significantly more likely to offer best practice smoking cessation care.

**Conclusion:**

Aboriginal Health Workers/Practitioners and Aboriginal Community Controlled Health Organizations play a critical role in delivering high quality, evidence based and culturally safe care to Aboriginal and Torres Strait Islander people. Aboriginal Health Workers/Practitioners are well placed to offer smoking cessation care. Ongoing funding and implementation of a targeted smoking cessation workforce with appropriate training and resources are urgently required.

**Implications:**

Aboriginal Health Workers/Practitioners are well placed to offer culturally safe, best practice smoking cessation care. However, due to the magnitude and complexity of Aboriginal Health Workers/Practitioners roles, it is challenging for smoking cessation care to be consistently and feasibly integrated into usual care. Acknowledging Australia’s National Preventative Health Strategy target of 27% or less Aboriginal and Torres Strait Islander people smoking by 2030, urgent investment and resourcing must be directed to building a skilled workforce to support quitting and maintaining smokefree behaviors, ensuring equitable access to smoking cessation care for Aboriginal and Torres Strait Islander peoples.

## Introduction

The rate of daily smoking among Aboriginal and Torres Strait Islander adults has dropped 9.8 percentage points since 2002.^1^ However, due to the ongoing effects of colonization, these rates continue to be disproportionately higher compared to other Australians with 40% of Aboriginal and Torres Strait Islander adults smoking daily in 201819.^2,3^ Smoking rates, factors and associations with smoking are diverse across Aboriginal and Torres Strait Islander people and communities.^1,4,5^ Commercial tobacco smoking is the leading preventable cause of morbidity and mortality for Aboriginal and Torres Strait Islander people.^3^ While reducing the rates of commercial tobacco use among Aboriginal and Torres Strait Islander people is included in nearly every health; plan,^6^ monitoring framework^7^ and strategy,^8^ it is seldom discussed or addressed in the context of coloniality to which it became available, embedded and sustained in Indigenous communities globally.^9^

Tobacco control evaluations have found that Aboriginal and Torres Strait Islander people are more likely than the general population to make a quit attempt, but less likely to succeed.^10^ Quit attempts are often short term and unsupported.^11^ This evidence suggests that while motivation to quit among Aboriginal and Torres Strait Islander people is high, it is possible that support for smoking cessation care (SCC) may not be appropriate, meaningful, and/or culturally safe. SCC is primarily offered through primary health settings. Standards for SCC are informed by evidence commonly drawn from the general population, notably that behavioral counseling and pharmacotherapies increase the rate of successful quitting^12^ and may not represent an appropriate evidence base for Indigenous peoples. The Royal Australian College of General Practitioners (RACGP) recommends pharmacotherapy [nicotine replacement therapy (NRT), varenicline, or bupropion] in combination with behavioral support, for all people during their quit attempt. For all Aboriginal and Torres Strait Islander people who smoke tobacco products, the guideline also recommends combination NRT accompanied by behavioral counseling.^13^ However, research has shown that Aboriginal and Torres Strait Islander people are less likely to have ever tried NRT^14^ with barriers of negative attitudes and beliefs toward NRT (ie, *“I prefer not to use medications”*) common among Aboriginal and Torres Strait Islander women.^15^ Little is known about the uptake and adherence to what is currently recommended as best practice SCC among Aboriginal and Torres Strait Islander peoples. Similarly, limited evidence is reported on effective strategies for SCC among Indigenous peoples internationally. However, consistent with the World Health Organization’s Framework Convention on Tobacco Control, calls have been made for targeted and tailored approaches for, by and with Aboriginal and Torres Strait Islander peoples to support effective smoking cessation interventions.^16^

Evidence on factors that influence healthcare professionals’ provision of SCC to Indigenous populations is scarce. However, international evidence reports a range of factors that influence healthcare professionals provision of SCC including lack of knowledge, lack of healthcare-specific smoking treatment policies,^17^ perceived role for providing smoking cessation care,^18^ lack of training and lack of time.^19,20^ In Australia, these factors have also been reported to influence low rates of NRT provision (4.7%),^21^ including among pregnant women.^22,23^

Studies have shown that providing education and training can improve health professionals’ knowledge, skills, and confidence in providing SCC and increase health care providers offer of cessation support, and in turn patients’ cessation rates.^24,25^ Smoking cessation training is also found to improve referral by health care providers to cessation services, including to the Quitline,^26^ a free telephone based SCC counseling service offered across Australia.

Aboriginal Health Workers (AHW) and Practitioners (AHP) play important roles in providing culturally safe care to Aboriginal and Torres Strait Islander people.^8^ However, research has reported multi-level barriers AHW face in quitting smoking themselves. This includes personal, social, and environmental stressors,^27,28^ as well as the broad range of skills, roles and AHW responsibilities, such as clinical, health promotion, education, and leadership responsibilities.^29^ In 2021, there were 17 Registered Training Organizations (RTO’s) to teach qualification that an Aboriginal and Torres Strait Islander Health Worker (Cert II, III and IV, Diploma and Advanced Diploma in Aboriginal and/or Torres Strait Islander Primary Health Care) and Aboriginal and Torres Strait Islander Health Practitioner (Certificate IV and Diploma in Aboriginal and/or Torres Strait Islander Primary Health Care Practice) are required to undertake.^30^ The National Aboriginal and Torres Strait Islander Health Workers and Practitioners Association (NAATSIHWP) is the peak body for Aboriginal and Torres Strait Islander health workers and practitioners. NAATSIHWP defines an Aboriginal and/or Torres Strait Islander Health Worker as *“An Aboriginal and/or Torres Strait Islander person who has gained a Certificate II or higher qualification in Aboriginal and/or Torres Strait Islander Primary Health Care from one of the health trainings packages”.*^31^ And an Aboriginal and/or Torres Strait Islander Health Practitioner as *“An Aboriginal and/or Torres Strait Islander person who has gained a Certificate IV in Aboriginal and/or Torres Strait Islander Primary Health Care Practice and has successfully applied for and been registered with the Aboriginal and Torres Strait Islander Health Practice Board of Australia through the Australian Health Practitioner Regulation Agency (AHPRA)”*.^28^ At the time of writing, formal SCC training is not readily available to AHW/P, nor is SCC training a required component to meet registration criteria. There are currently 1052 AHW/AHP nationally who are registered with NAATSIHWP, working in both Aboriginal Community Controlled Health Organizations and mainstream health services across the country.

AHW/Ps are often the first contact Aboriginal and Torres Strait Islander people have with a health service and have played a critical role in improving the cultural safety of mainstream healthcare settings. Although AHW/Ps have been identified as best placed to offer culturally safe and appropriate smoking cessation care to Aboriginal and Torres Strait Islander people, there is no funding (either through Medicare or block funding) for AHW/P to provide SCC. A recent environmental scan of tobacco programs in New South Wales found that many Aboriginal Community Controlled Health Organizations delivered tobacco control programs unfunded, with significant work undertaken in smoke-free policies, health promotion and education but there was limited one-on-one or group-based smoking cessation support.^32^ While guidelines for SCC are in place for general practitioners, and funding available through Medicare for these services, including telehealth,^33^ there are currently no cessation guidelines or cessation funding specifically for AHW/P.

This paper is part of the *Which Way?* study which aims to develop an Indigenous-led evidence base for SCC to support Aboriginal and Torres Strait Islander women to be smoke-free. The details of the project have been reported elsewhere.^34^ This study was informed by the recent *Which Way?* national survey of Aboriginal and Torres Strait Islander women on smoking and quitting, 64% of whom requested smoking cessation support from their AHW/P.^35^

This study aims to understand the knowledge, attitudes and practices of AHW/P to inform appropriate policies and practices for SCC to support and empower Aboriginal and Torres Strait Islander people to be smoke-free. This study was co-designed, analyzed and reported in partnership with NAATSIHWP and Aboriginal communities in urban and regional New South Wales, Australia.

## Methods

### Research Team

This project is informed, implemented, and reported by an Indigenous-led research team, we recognize that our lived experience and worldviews influence the way that the study was conducted^36–38^ which is central to developing an Indigenous-led evidence base for SCC. The study was conceptualized and led by the first author (MK, Wiradjuri woman), in partnership with NAATSIHWP and Aboriginal communities represented by: HL, SB, PH, KB. Our team brings Aboriginal and Torres Strait Islander lived experience (MK, KB, HL, SB), Indigenous lived experience (RM), expertise in Aboriginal health services (PH, HL, SB), Aboriginal Health Workers (HL, SB, KB), Indigenous tobacco research (AM, MK, RM, YBZ) and epidemiology (RM, YBZ).

This work was led by the interest and needs of Aboriginal and Torres Strait Islander people and governed by the *Which Way?* Aboriginal Governance Committee, inclusive of partnering communities.^34^ The *Which Way?* study utilized integrated knowledge translation, embedding knowledge users, to ensure Aboriginal and Torres Strait Islander community and healthcare relevance with a balance of Indigenous knowledge and wisdom with scientific excellence.

### Design and Procedure

An online cross-sectional survey was conducted from June to September 2021 targeting AHW and AHP across Australia. The survey was promoted via email invitation to all registered members (*n* = 1,052) of NAATSIHWP and through the National Best Practice Unit, Tackling Indigenous Smoking. The sample was sourced from the NAATSIHWP members mailing list of Aboriginal and Torres Strait Islander health workers and practitioners. An invitation email was sent to all members from NAATSIHWP email (*n* = 1,052). Further promotion of the study was done through the National Best Practice Unit, Tackling Indigenous Smoking through their newsletter. Participants were deemed eligible if they were (1) an Aboriginal Health Worker or Aboriginal Health Practitioner; and (2) identified themselves as Aboriginal, Torres Strait Islander, or Aboriginal and Torres Strait Islander.

### Measures

Survey items were informed by the literature and Aboriginal knowledge through *Which Way?* partners, in particular three female AHW in management roles at partnering services, and NAATSIHWP. Validated measures and an iterative co-design process consisted of Aboriginal researchers and Aboriginal community partners informing the survey item categories, which were developed to understand the knowledge, attitudes, and practices of AHW/P toward SCC. SCC practice questions included clinical guidelines,^13^ Australian Government Department of Health and Aged Care suggested methods,^39^ and Aboriginal community practice.^40^ The preliminary survey was then reviewed by AHWs at our partnering services to establish face and content acceptability, validity, and feasibility, before pilot testing with 10 community members.

Sociodemographic variables included Aboriginal and/or Torres Strait Islander status (Aboriginal, Torres Strait Islander, and both Aboriginal and Torres Strait Islander), age (continuous), gender, smoking status (never smoker, ex-smoker, and smoker; recategorized to current smoker yes/no), and state/territory of residence was elicited through a single select dropdown option.

Professional work experience was collected as a continuous variable, and then categorized to less than 5 years, 510 years, and more than 10 years of experience. Participants’ specific role in their workplace was asked in an open text question. Additionally, the type of workplace was classified into two categories: 1) either Aboriginal Community Controlled Health Organization(s), and 2) any other workplace, including General Practice.

Training in smoking cessation support (“yes”/“no”) was used as a proxy for knowledge. Attitude towards the provision of SCC was measured using one yes/no statement regarding participants’ belief of whether they perceive providing smoking cessation support is part of their role.

### Outcomes

The primary outcomes of interest were the practice of the three main components of SCC—1) any form of counseling; 2) recommending, or providing a combination of forms of NRT; and 3) referral to Quitline/specialist service. This was measured used a Likert scale: never to always, and re-categorized into three groups as never, sometimes (occasionally and sometimes), and always/often (often and always).

### Statistical Analysis

Participants were excluded from analyses if they were not eligible to be in the study or did not provide consent. Descriptive statistics using frequency and percentage tables were conducted for SCC components and all other categorial variables; and mean and standard deviation (SD) for the continuous variable.

Bivariate multinomial logistic regression was conducted separately for each component of SCC and various factors. The reference category for the outcome variables was “never” providing the above-mentioned components of SCC. Predictor variables that showed significant association in the bivariate logistic regression were included in the multivariate multinomial logistic regression. Findings were reported using Adjusted Odds Ratios (AOR) and 95% Confidence Intervals (CI). An alpha level of 0.05 was the threshold for statistical significance. Data analysis was carried out using SPSS statistical software version 27.0.

### Ethics

The *Which Way?* study was developed by Aboriginal and Torres Strait Islander communities in New South Wales, Australia. The project upholds ethical principles of research with Aboriginal and Torres Strait Islander peoples in line with the National Health and Medical Research Council Guidelines for ethical conduct in Aboriginal and Torres Strait Islander Health Research, the Aboriginal Health and Medical Research Council’s (AH&MRC) Ethical Guidelines: Key Principles (2020), and the international CONSIDER statement.^41^ Ethics approvals included AH&MRC (#1603/19) and The University of Newcastle (#H-2020-0092). All participants provided informed consent. At the end of the survey interested participants could offer their email address and go in the draw for one iPad.

## Results

Of the 1052 registered NAATSIHWP members, the survey was initiated by 379 participants. Of the 319 who consented, two were deemed ineligible as they were not Aboriginal and/or Torres Strait Islander, with 256 completing the survey (24.3% response rate, completion rate 67.5%). The mean age of participants was 44.98 ± 12 years. The majority of participants (78.9%) were female, 44.4% of participants were from NSW, and 75.4% were nonsmokers as detailed in Table 1.

**Table 1:** Socio-demographic and Work Related Characteristics of Aboriginal Health Workers and Practitioners in Australia (*n *= 256).

Variable name	Frequency (n)	% (95%CI)
Age (mean ± SD)	44.98 ± 12 years old	43.4‐46.4 years old
Gender		
**Male**	50	19.5(15.4‐25.3)
**Female**	202	78.9(73.7‐83.8)
**Others**	2	0.8(0.2‐3.1)
State		
**NSW**	112	44.4(38.4‐50.6)
**Victoria**	20	7.9(5.1‐12)
**Queensland**	67	26.6(21.4‐32.4)
**South Australia**	22	8.7(5.8‐12.9)
**Western Australia**	18	7.1(4.5‐11)
**Tasmania**	3	1.2(0.3‐3.6)
**Northern Territory**	10	4(2.1‐7.2)
Aboriginality status		
**Yes, Aboriginal**	223	87.1(82.3‐90.7)
**Yes, Torres Strait Islander**	19	7.4(4.7‐11.3)
**Yes, both Aboriginal and Torres Strait Islander**	14	5.5(3.2‐9)
Smoking status		
**Smoker**	62	24.6(19.6‐30.3)
**Non-smoker**	190	75.4(69.6‐80.3)
Position		
**Aboriginal Health Worker (including in training)**	117	45.7(39.6‐51.8)
**Aboriginal Health Practitioner (including in training)**	129	50.4(44.2‐56.5)
Others*	10	3.9(2.1‐7.1)
Professional work experience		
**Less than 5 years**	60	23.5(18.7‐29.1)
**5–10 years**	68	26.7(21.5‐32.4)
**10+ years**	127	49.8(43.6‐55.9)
Specialty		
**No**	121	47.8(41.7‐54)
**Yes**	132	52.2(45.9‐58.2)
Duties of the current role		
Cultural support/advocacy	215	84(78.9‐88)
Health Promotion/Education	214	83.6(78.5‐87.6)
Community support	167	65.2(59.1‐70.8)
One on one appointments	161	62.9(56.7‐68.6)
Making referrals	160	62.5(56.3‐68.2)
Support community with health appointments	146	57(50.8‐62.9)
Data collection	139	54.3(48.1‐60.3)
Brief intervention programs	136	53.1(46.9‐59.1)
Report data	128	50(43.8‐56.1)
Case Management	123	48(41.9‐54.1)
Provide cultural supervision to staff	122	47.7(41.5‐53.8)
Conducting assessments	116	45.3(39.2‐51.4)
Aboriginal Liaison	114	44.5(38.5‐50.7)
Community transport	111	43.4(37.3‐49.5)
Group sessions	110	43(37‐49.1)
Triage	108	42.2(36.2‐48.3)
Crisis support	96	37.5(31.7‐43.6)
Counselling	75	29.3(24‐35.1)
Intake	73	28.5(23.2‐34.3)
Assist in developing of health management plans	66	25.8(20.7‐31.5)
Committee presentation	65	25.4(20.4‐31.1)
Other	21	8.2(5.3‐12)
Workplace		
**ACCHO**	144	57.6(51.3‐63.6)
**General Practice**	106	42.4(36.3‐48.6)

ACCHO—Aboriginal Community Controlled Health Organisation, * Others include Aboriginal Midwife Practitioner, Aboriginal Senior Health Worker Isolated Practice Area, Aboriginal team leader community.

Among the participants, 23.5% had less than 5 years work experience, 26.7% had 5 to 10 years, and 49.8% had more than 10 years. Approximately half (50.4%) of participants were AHP, and 45.7% were AHWs. Half of the participants (52.2%) specialized in a particular field such as mental health. The majority of participants (84%) provided cultural support, 83.6% provided health education, 65.2% provided community support, and 47.7% provided cultural supervision for staff and others. Just above half (57.6%) of the participants worked at Aboriginal Community Controlled Organizations.

Among the participants, 76.5% of AHW/P believed providing SCC was part of their role. The majority (62.8%) of AHW/P reported being trained in SCC.

### Practice of SCC

Provision of the different components of SCC is described in Table 2. Smoking cessation counseling was always/often provided by 41.9% of the AHW/Ps, provided some of the time by 42.4% of the AHW/Ps, and never provided by 12.9% of the AHW/Ps. A combination of NRT oral forms and patches was offered always/often by 23.1% of the AHW/Ps. One in five (22.2%) AHW/Ps never offered combination NRT when supporting smokers in their quit attempt. Quitline referral was offered always/often by 44.9% of the AHW/Ps, 38.8% some of the time and never offered by 12.5% of the AHW/Ps.

**Table 2:** Practices of SCC among Aboriginal Health Workers and Practitioners in Australia (*n *= 256).

Variable name	Never	Occasionally	Sometimes	Often	Always
**Counselling**	28 (12.9%)	30 (13.8%)	62 (28.6%)	55 (25.3%)	36 (16.6%)
**Recommend NRT combination**	48 (22.2%)	29 (13.4%)	32 (14.8%)	46 (21.3%)	50 (23.1%)
**Refer to Quitline/specialist service**	27 (12.5%)	34 (15.7%)	50 (23.1%)	47 (21.8%)	51 (23.6%)

### Factors Associated With SCC Practices

Table 3 provides the results of the multivariate logistic analysis for the different components of SCC.

**Table 3:** Multivariate nominal logistic regressions of the provision of various smoking cessation care components practices among Aboriginal Health Workers and Practitioners in Australia (n* *= 256).

Factors	Counselling AOR (95%CI)		Combination NRT AOR (95%CI)		Quitline Referral AOR (95%CI)	
	Sometimes	Always/Often	Sometimes	Always/Often	Sometimes	Always/Often
SCC Training						
**Yes**	3.51 (1.31‐9.44)*	4.11 (1.46‐11.55)**	5.24 (2.15‐12.76)**	7.59 (3.11‐18.54)**	3.16 (1.10‐9.06)*	4.15 (1.45‐11.87)**
Role SCC						
**Yes**	4.44 (1.65‐11.94)**	12.19 (4.02‐36.73)**	2.65 (1.06‐6.59)*	8.65 (3.11‐24.0)**	3.48 (1.19‐10.20)*	7.76 (2.53‐23.82)**
Smoking status						
**Smoker**	Ref	Ref	Ref	Ref	Ref	ref
**Non-smoker**	0.87 (0.31‐2.43)	1.87 (0.62‐5.62)	0.40 (0.15‐1.05)	0.46 (0.17‐1.21)	0.46 (0.14‐1.49)	0.75 (0.23‐2.46)
Experience						
**>10 years**	1.42 (0.46‐4.39)	2.35 (0.72‐7.73)	1.79 (0.60‐5.35)	1.72 (0.59‐4.98)	3.92 (1.22‐12.64)*	2.26 (0.70‐7.26)
**5–10 years**	1.82 (0.42‐7.91)	1.69 (0.37‐7.79)	0.68 (0.19‐2.33)	0.52 (0.16‐1.69)	1.59 (0.37‐6.88)	1.99 (0.49‐8.15)
**<5 years**	Ref	Ref	Ref	Ref	Ref	ref
ACCHO						
**ACCHS**	1.81 (0.66‐4.95)	2.28 (0.80‐6.46)	1.20 (0.50‐2.88)	2.68 (1.12‐6.43)*	0.29 (0.09‐0.93)*	0.39 (0.12‐1.29)
Other*	Ref	Ref	Ref	Ref	Ref	ref

The reference category is “Never” providing the smoking cessation component; ACCHS—Aboriginal Community Controlled Health Service; Ref-reference category; All of the time—always and often; Some of the time—sometimes/occasionally; *P-value < 0.05; **P-value < 0.01.

Other includes any other healthcare practices such as general practices.

Receiving smoking cessation training and believing that providing SCC is part of their professional role were both significantly associated with the provision of all the smoking cessation care components (Table 3). Working at Aboriginal Community Controlled Health Services (ACCHO) was significantly associated with always/often providing combination NRT (AOR 2.68 95% CI 1.12–6.43) and sometimes referring to the Quitline (AOR 0.29 95% CI 0.09–0.93). AWH/P smoking status was not associated with the provision of SCC components. Having a professional work experience of more than 10 years was positively associated with Quitline referral (AOR 3.92 95% CI 1.22–12.64).

## Discussion

This study is among the first Indigenous-led studies to report the AHW/P practices in providing SCC to the Aboriginal and Torres Strait Islander community. This study, conducted in partnership with NAATSIHWP, presents novel evidence from a quarter (24.3%) of the current AHW/P workforce.

AHW/P workforce is experienced with nearly half of respondents reporting over 10 years of work in their role. This included over half having a speciality and holding practitioner registration, and all participants reporting a broad range of duties required to undertake their role. AHW/P’s in our study work in Aboriginal Community Controlled Health Organizations (ACCH) (57.6%) and mainstream health services (42.4%) with an almost even divide between the two. A novel finding from this study was that AHW/P experience (time in the workforce) and working at an ACCHO was positively associated with offering combination NRT.

While research in this setting has previously reported the possibility of AHW/P smoking status as a barrier to offering SCC to the community,^42^ this was not evidenced in our sample. Previous research reported smoking rates among AHW were only modestly lower than the broader Aboriginal and Torres Strait Islander population.^43^ However our study found much lower smoking rates compared to the broader Aboriginal and Torres Strait Islander population (24.6% vs. 40.2%).^1^ Reports demonstrate successful reductions in smoking rates in the Aboriginal and Torres Strait Islander community.^1^ Our findings support this and provide further evidence that smoking status is not associated with any practice of SCC.

Despite calls being made by Aboriginal and Torres Strait Islander people for over a decade,^44^ there is no specific training or funding for AHW/P in regard to SCC. However, to date, no study has specifically asked the workforce how they perceive their role in reference to SCC. We found that a large proportion of AHW/P (76.5%) did feel that providing SCC is part of their role and this attitude was positively associated with offering SCC. This is in line with previous research which also found that attitudes towards SCC to be significantly associated with practice.^45^ As such, this study reports additional strengths among AHW/P to contribute to accelerating reductions in smoking among Aboriginal and Torres Strait Islander people.

At the time of writing, there is no formal SCC training as part of AHW/P qualification and registration requirements. However, our study shows that receiving some level of training was found to influence SCC offered which included counseling, combination NRT and Quitline referrals. The importance and impact of training to improve the provision of SCC is well documented across the globe,^19,46^ including in Aboriginal and Torres Strait Islander maternal health settings.^47^ This evidence suggests that the development and implementation of nationally accredited smoking cessation training for AHW/P could significantly impact high quality, evidence-based care provided to Aboriginal and Torres Strait Islander people to quit smoking. This training must be developed in partnership with AHW/P such as NAATSIHWP to ensure relevance to this specific workforce. Acknowledging the National Preventative Health Strategy target of 27% or less Aboriginal and Torres Strait Islander people smoking by 2030, urgent investment and resourcing must be directed to building a skilled workforce to support quitting and maintaining smokefreebehaviors.^8^

While AHW/P who worked at an ACCHO were less likely to offer community members a referral to Quitline, they were significantly more likely to offer combination NRT. It is important to highlight that Aboriginal and Torres Strait Islander people’s use of Quitline has not been evaluated. A South Australian Quitline study found Aboriginal and Torres Strait Islander callers received significantly fewer call backs and were less likely to set a quit date compared to non-Aboriginal callers.^48^ While international research has engaged with Indigenous peoples use of Quitline services,^49^ further research is warranted to understand Aboriginal and Torres Strait Islander experiences of the Quitline across Australia.

### Implications for Policy and Practice

The Australian Government recently launched the first National Aboriginal and Torres Strait Islander Health Workforce Strategic Framework and Implementation Plan 2021–2031.^50^ The Plan’s Vision is for *Aboriginal and Torres Strait Islander peoples [to] enjoy long, healthy lives that are centered in culture, with access to services that are prevention-focussed, responsive, culturally safe and free of racism and inequity; and aims to increase the Aboriginal and Torres Strait Islander workforce across all roles and locations*.^50^ The government has also invested a further $187.8 million in the successful Tackling Indigenous Smoking Programme^51^ which will focus on population health activities. While these investments are warranted and welcomed, neither will specifically impact changes or improvements to Aboriginal and Torres Strait Islander people receiving SCC.

One approach to impacting the provision of SCC is an expansion of Medicare item numbers specifically for AHW/P to ensure coverage under Australia’s universal health insurance scheme (Medicare). ACCHOs are required to utilize activity-based funding models for the provision of care, and our study found that AHW/P in ACCHO were more likely to provide evidence-based care. However, this is often delivered unfunded or underfunded in an ACCHO setting^32^ which is both unsustainable and imposes additional burden on Aboriginal and Torres Strait Islander communities. Last year the Medicare Benefits Schedule Review Taskforce overruled the recommendations made by the Aboriginal and Torres Strait Islander Project Reference Group for an expansion of Medicare item numbers.^52^ The expansion of numbers would have allowed Aboriginal and Torres Strait Islander people to have increased follow up by AHW/P as well as allied health and nurses which would support culturally safe care and services to generate necessary financial support. Both of which are critical for the National Aboriginal and Torres Strait Islander Health Workforce Strategic Framework and Preventative Health Strategy. Appropriate and sustainable funding models to enhance the provision of SCC are urgently required and should consider both Medicare item numbers and block funding models. With reductions targets in place for Aboriginal and Torres Strait Islander people by 2030, it is critical that Australia does not replicate the short comings of the Closing the Gap strategy.^7^ Namely not aligning health targets with appropriate resourcing and leadership by and for Aboriginal and Torres Strait Islander peoples.^53^

The reformed Closing the Gap agenda recognizes the importance of Aboriginal and Torres Strait Islander leadership to achieve improved health outcomes for Aboriginal and Torres Strait Islander people, and in particular the importance of our Aboriginal Community Controlled Sector.^7^ This is aligned with the World Health Organizations Framework Convention on Tobacco Control and the rights of Indigenous peoples in the *development, implementation and evaluation of tobacco control programs that are socially and culturally appropriate to their needs and perspectives.*^54^ These must be upheld when addressing policy and practice reform to accelerate reductions in Aboriginal and Torres Strait Islander smoking prevalence.

## Conclusion

Australia is a party to the Framework Convention on Tobacco Control which addresses the critical importance of Indigenous peoples and communities’ engagement in the development, implementation and evaluation of tobacco control. AHW/P and ACCHO play a critical role in delivering high quality, evidence based and culturally safe care to Aboriginal and Torres Strait Islander people. AHW/P are well placed to support Aboriginal and Torres Strait Islander peoples to quit smoking and promote smokefree behaviors. However, acknowledging the complexity and multitude of AHW/P roles, the time required to offer comprehensive SCC, it is challenging to expect that feasible, best practice, SCC can be integrated into usual care. AHW/P that received training, felt SCC was part of their role and were based in ACCHO’s were significantly more likely to offer best practice SCC. Improvements can be made to the provision of evidence-based SCC which has great potential to enhance the health and wellbeing of Aboriginal and Torres Strait Islander peoples. Ongoing funding and implementation of a targeted smoking cessation workforce, with appropriate training and resources should be a national priority.

## Supplementary Material

A Contributorship Form detailing each author’s specific involvement with this content, as well as any supplementary data, are available online at https://academic.oup.com/ntr.

ntac256_suppl_Supplementary_Taxonomy-form

## Data Availability

Data not publicly available.
